# Efficacy and Safety of TACE Combined with Regorafenib versus TACE in the Third-Line Treatment of Colorectal Liver Metastases

**DOI:** 10.1155/2022/5366011

**Published:** 2022-12-03

**Authors:** Haohao Lu, Chuansheng Zheng, Li Fan, Bin Xiong

**Affiliations:** ^1^Department of Radiology, Union Hospital, Tongji Medical College, Huazhong University of Science and Technology, Jiefang Avenue No. 1277, Wuhan 430022, China; ^2^Hubei Province Key Laboratory of Molecular Imaging, Wuhan 430022, China; ^3^Department of Oncology, Union Hospital, Tongji Medical College, Huazhong University of Science and Technology, Jiefang Avenue No. 1277, Wuhan 430022, China

## Abstract

**Background:**

The liver is the most common site of metastasis in colorectal cancer. In patients with unresectable colorectal liver metastases, the 5-year survival rate is less than 5%. Many patients with colorectal liver metastases require effective subsequent therapy after the failure of standard first-line/second-line therapy. The purpose of this study is to investigate the efficacy and safety of TACE combined with Regorafenib versus TACE in the third-line treatment of patients with colorectal liver metastases.

**Method:**

The clinical data of 132 patients with colorectal liver metastases were collected. There were two groups: TACE + Regorafenib group (*N* = 63); TACE group (*N* = 69). TACE uses CalliSpheres® drug-loaded microspheres (loaded with irinotecan). Regorafenib is administered at a dose of 120 mg once daily. If the patient is severely intolerable, the regorafenib dose is adjusted to 80 mg once daily. Primary study endpoints were (1) to evaluate the tumor response, ORR, and DCR and (2) to evaluate OS and PFS in the two groups. Secondary study endpoints were (1) to compare the performance status, CEA, CA19-9 after treatment between the two groups and (2) to compare the incidence of adverse events between the two groups.

**Results:**

There were significant differences in tumor response, ORR, DCR, OS, and PFS after treatment between the two groups. TACE combined with the Regorafenib group versus the TACE group: ORR (57.1% vs 33.3%), DCR (82.5% vs 68.1%), mOS (18.2 months vs 11.3 months), and mPFS (8.9 months vs 5.3 months). The performance status after treatment was better in the TACE + Regorafenib group than in the TACE group (*P* < 0.05). The CEA and CA19-9 negative rates after treatment were higher in the TACE + Regorafenib group than in the TACE group (*P* < 0.05).

**Conclusion:**

For the third-line treatment of colorectal liver metastases, the combination of TACE + Regorafenib had better tumor response, OS, and PFS than TACE TACE + Regorafenib combination could be considered as salvage therapy for colorectal liver metastases who failed the first- and second-line standard therapy.

## 1. Introduction

Globally, colorectal cancer is a malignant tumor with the second highest incidence and the third highest mortality [1], which seriously threatens human life and health. The liver is the most common site of metastasis in colorectal cancer [2, 3]. Colorectal liver metastases (CRLM) include synchronous liver metastases and metachronous liver metastases. Liver metastasis occurs at the same time or within 6 months after the diagnosis of colorectal cancer, which belongs to synchronous liver metastasis; liver metastasis occurs more than 6 months after the diagnosis of colorectal cancer, which belongs to metachronous liver metastasis [4]. Studies have reported that about 50% of colorectal cancer patients may have liver metastases during the course of the disease. Of these, 15–25% have synchronous liver metastases [5]. Even after achieving curative resection of the primary tumor of colorectal cancer, the chance of developing metachronous liver metastases is as high as 30% [3]. Liver metastasis from colorectal cancer is the leading cause of death [6]. The survival time of patients with untreated colorectal liver metastases was 6.9 months. Approximately 35% of patients with colorectal cancer have metastases confined to the liver alone and become the ultimate cause of death in approximately 50% of these patients [7, 8]. Studies have reported that when colorectal cancer patients develop liver metastases, only 10–20% of patients can be treated with radical surgery [9], and their 5-year survival rate is around 35–55% [10]. However, in patients with unresectable CRLMs, the 5-year survival rate is less than 5%.

For patients with unresectable colorectal liver metastases, a two-drug or three-drug chemotherapy regimen of 5-FU (or capecitabine) combined with oxaliplatin and/or irinotecan, combined with targeted medicine, is a standard first- and second-line treatment regimen and can significantly improve the prognosis of patients [10]. However, many patients with CRLMs experience intrahepatic disease progression after first-line/second-line therapy and require continued treatment [11]. Clinical studies have found that colorectal liver metastases can be localized in the liver for a long time without extrahepatic metastases [12], so the treatment strategy for colorectal liver metastases should be to actively implement effective local treatment under the premise of multidisciplinary treatment, such as ablation procedures [13], regional hepatic intra-arterial chemotherapy [14], chemoembolization [15], radioembolization [16], and radiation therapy [17], including stereotactic radiation therapy.

Transcatheter arterial chemoembolization (TACE) is an effective treatment for primary liver cancer and liver metastases [18]. Its main principle is to inject chemotherapeutic drugs followed by embolic agents into blood vessels supplying nutrients to the tumor via a catheter. TACE treats liver cancer through the following mechanisms. First, the cytotoxicity of pharmacological drugs can induce the apoptosis of tumor cells and inhibit the proliferation of tumor cells. Second, after the embolization of tumor vessels, it leads to ischemia, hypoxia, and necrosis of tumor tissues [19]. According to the different embolic agents used in TACE, TACE consists of conventional TACE (C-TACE) and TACE with drug-eluting beads (DEB-TACE). Karanicolas et al. [20] reported that DEB-TACE appears to offer a survival benefit in the second-line treatment of the unresectable CRLMs. CalliSpheres® drug-loaded microspheres are a new type of embolization material. It has the following advantages: (1) Its component is PVA, which is a permanent embolic material. It can effectively embolize tumor blood vessels, leading to tumor ischemia, hypoxia, and necrosis. (2) When the microspheres reach the target vessel, they can slowly release the chemotherapy drugs they have been preloaded. It can maintain a high concentration of chemotherapy drugs in the tumor area for up to one month; meanwhile, the concentration of chemotherapy drugs in the peripheral blood is low, reducing the systemic toxicity of chemotherapy drugs [21]. However, hypoxia after TACE induces an increase in VEGF levels, leading to tumor recurrence and metastasis [22]. Regorafenib is a multitarget tyrosine kinase inhibitor. Regorafenib can inhibit a variety of kinases involved in angiogenesis (including lymphangiogenesis), cell proliferation, and tumor growth as well as the tumor microenvironment, mainly including VEGFR1∼3 (angiogenesis), KIT, RET (tumor formation), PDGFR-*β*, and FGFR (tumor microenvironment), thereby controlling tumor growth and delaying disease progression [23]. Regorafenib is recommended as a third-line treatment for patients with unresectable CRLM and beyond [24]. Mechanistically, the combination of TACE and Regorafenib has complementary effects and can compensate for the problems posed by the rise in VEGF levels after TACE [25]. Although some studies have reported that TACE combined with Regorafenib was more effective in patients with CRLM than Regorafenib alone [26], there has been no comparative study between TACE + Regorafenib and TACE alone in the treatment of CRLM. The aim of this study was to investigate the efficacy and safety of TACE combined with Regorafenib versus TACE in the third-line treatment of patients with colorectal liver metastases.

## 2. Materials and Methods

### 2.1. General Information

**Figure 1:**
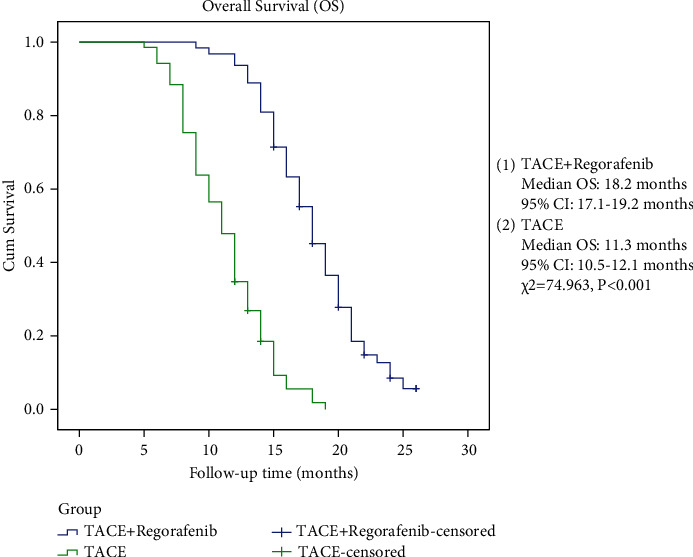
The patient selection process.

This was a single-center retrospective study. The clinical data of 132 patients with colorectal liver metastases admitted to the Department of Intervention Therapy, Union Hospital, Tongji Medical College, Huazhong University of Science and Technology, from January 2016 to December 2019 were collected. Inclusion criteria were (1) the primary tumor was confirmed as colorectal cancer by pathology; (2) the lesion was diagnosed as colorectal liver metastasis by pathology or imaging examination; (3) there was no metastasis to other organs than the liver; (4) the lesion progressed after standard first- and second-line treatment; (5) liver function: Child-Pugh class A or B, performance score (ECOG) 0–2; (6) intrahepatic lesions had not been treated with TACE before; and (7) estimated survival time ≥3 months; and (8) age 18–70 years old. Exclusion criteria were (1) liver function: Child-Pugh class C, physical score (ECOG) > 2; (2) lesions more than 60% of the liver volume; (3) severe coagulopathy and cannot be corrected; (4) combined heart, lung, brain, kidney, and other serious dysfunction; (5) pregnant or lactating patients; (6) allergic to a contrast agent or Regorafenib; (7) the patient was not followed up on time according to the doctor's advice, or the follow-up examination data were incomplete. The patients were divided into two groups: TACE + regorafenib group (*N* = 63); TACE group (*N* = 69) (Figure 1). The baseline data of patients including gender, age, location of the primary tumor, whether the primary tumor was surgically resected, Child-Pugh classification of liver function, ECOG score, ALT, AST, total bilirubin, CEA, CA19-9, number and the maximum diameter of lesions were collected.

#### 2.1.1. Child-Pugh Classes

Patients were scored for hepatic encephalopathy, ascites, total bilirubin, albumin, and prolonged prothrombin time. Grade A is 5 –6 points, grade B is 7–9 points, and grade C is ≥ 10 points.

### 2.2. Method

#### 2.2.1. Treatment Procedures

The patient was placed in a supine position, and the inguinal region was disinfected and draped. Local anesthesia was performed after the injection of 1% lidocaine at the femoral artery puncture site. The femoral artery was accessed using the Seldinger technique, and a 5F vascular sheath was placed. The 5 F Yashino catheter was inserted into the celiac trunk and superior mesenteric artery through the vascular sheath, and arteriography was performed to identify the feeding artery of the tumor. A 2.7 F microcatheter was then superselectively cannulated into the tumor-feeding artery and injected with CalliSpheres® drug-loaded microspheres, and the embolization endpoint was that the blood flow in the tumor-feeding artery was close to the stagnant state. Patients received a reexamination of their enhanced CT or MRI, CEA, and CA19-9 every 3 –4 weeks. According to reexamination conditions, it was decided whether TACE should be performed again. CalliSpheres® drug-loaded microspheres with 100–300 *µ*m particle sizes were selected. CalliSpheres® drug-loaded microspheres were first aspirated and then allowed to stand, and the supernatant was discharged after the microspheres sank; 120 mg irinotecan was dissolved using 5 ml of the vehicle and loaded into a bottle of CalliSpheres® drug-loaded microspheres for 30 min [27]. Nonionic contrast medium was mixed 1 : 1 with CalliSpheres® Solution before injection, and then slowly injected through the catheter at an injection rate of 1 mL/min to maintain forward blood flow during the injection. CalliSpheres® were administered according to intrahepatic lesions. Embolization endpoints were near the stasis of forward flow in the target vessel. Once the embolic endpoint is reached, the injection is stopped, whether or not the embolic agent is completely injected. If the embolic endpoint is still not reached after injection of a vial of CalliSpheres®, blank microsphere (8Spheres®) may be supplemented. If it is necessary to supplement blank microspheres 8Spheres®, select 300–500 *µ*m particle size.

Regorafenib is administered at a dose of 120 mg once daily for three weeks, followed by a one-week break. If the patient is severely intolerable due to adverse drug reactions, the Regorafenib dose is adjusted to 80 mg once daily. In the TACE combined with the Regorafenib group, Regorafenib was temporarily discontinued 3 days after each TACE treatment.

### 2.3. Outcome Measures

#### 2.3.1. Primary Study Endpoints

1. Patients in both groups were evaluated for tumor response after 3 months of treatment using mRECIST criteria, including complete response (CR), partial response (PR), stable disease (SD), and progressive disease (PD)
2. To evaluate the objective response rate (ORR), and disease control rate (DCR) of the two groups
3. To evaluate OS and PFS in the two groups

#### 2.3.2. Secondary Study Endpoints

1. Changes in liver function and performance status (ECOG score) before and after treatment in the two groups
2. The changes in tumor markers (CEA, CA19-9) before and after treatment in the two groups
3. The incidence of adverse events after treatment in the two groups

The patients were compared before treatment and at the time of reexamination after 3 months of treatment.

ORR: Proportion of patients achieving CR + PR in total patients after 3 months of treatment.

DCR: Proportion of patients achieving CR + PR + SD in total patients after 3 months of treatment.

PFS: It refers to the time from the initiation of treatment to the occurrence of tumor progression or death.

### 2.4. Statistical Methods

Statistical analysis was performed using the SPSS 24.0 software. Enumeration data were expressed as the number of cases (percentage), and a chi-square test was used for differences, including Pearson chi-square and Fisher's exact test. Measurement data were expressed as mean ± standard deviation, and two independent samples *t*-test was used. OS and PFS were shown by Kaplan –Meier curves, and the log rank test was used to compare OS and PFS between the two groups. *P* < 0.05 was considered to indicate a statistically significant difference.

## 3. Results

### 3.1. Basic Information

1. Comparison of pretreatment enumeration data between the TACE + Regorafenib group and the TACE group (Table 1).
2. There were no statistically significant differences in gender, liver function grade, ECOG score, CEA, CA19-9, location of the primary tumor, whether the primary tumor was surgically resected, and size/number of liver metastases between the two groups.
3. Chi-square test was used for comparison between two groups with a*P* value > 0.05 and no statistical difference.
4. Comparison of pretreatment measurement data between the TACE + Regorafenib group and the TACE group (Table 1).

There was no statistically significant difference in age, ALT, AST, and total bilirubin before treatment between the two groups.

Comparisons between two groups were performed using the *t*-test with *P* value > 0.05 and no statistical difference.

### 3.2. Changes in Liver Function before and after Treatment in Both Groups

1. Comparison of liver function after treatment between the two groups (Table 2): ALT: 50.4 ± 23.2 U/L in the TACE + Regorafenib group and 47.2 ± 18.7 U/L in the TACE group; AST: 47.5 ± 17.9 U/L in the TACE + Regorafenib group and 46.6 ± 11.0 U/L in the TACE group; total bilirubin: 19.1 ± 4.8 *µ*mol/L in the TACE + Regorafenib group and 19.9 ± 4.1 *µ*mol/L in the TACE group; Comparisons between two groups were performed using the *t*-test with *P* value > 0.05 and no statistical difference.
2. Compare the changes in liver function before and after treatment in each group (Table 3).

#### 3.2.1. TACE + Regorafenib Group

ALT: before treatment, 38.3 ± 8.4 U/L; after treatment, 50.4 ± 23.2 U/L; (*P* < 0.05). AST: before treatment, 34.2 ± 9.7 U/L; after treatment, 47.5 ± 17.9 U/L; (*P* < 0.05). Total bilirubin: before treatment, 15.8 ± 5.6 *μ*mol/L; after treatment, 19.1 ± 4.8 *μ*mol/L; (*P* < 0.05).

#### 3.2.2. TACE Group

ALT: before treatment, 39.2 ± 10.5 U/L; after treatment, 47.2 ± 18.7 U/L; (*P* < 0.05). AST: before treatment, 32.7 ± 11.6 U/L; after treatment, 46.6 ± 11.0 U/L; (*P* < 0.05). Total bilirubin: before treatment, 14.1 ± 4.5 *μ*mol/L; after treatment, 19.9 ± 4.1 *μ*mol/L; (*P* < 0.05).

A paired sample *t*-test was used, and a *P* value < 0.05 was considered to indicate a statistical difference.

### 3.3. Incidence of Adverse Events after Treatment in the Two Groups (Table 4)

Fever: 41 (65.1%) in the TACE + Regorafenib group, 44 (63.8%) in the TACE group (*P* > 0.05); nausea and vomiting: 25 (39.7%) in the TACE + Regorafenib group, 28 (40.6%) in the TACE group (*P* > 0.05); abdominal pain: 43 (68.3%) in TACE + Regorafenib group, 22 (31.9%) in the TACE group (*P* < 0.05); hand-foot reaction: 31 (49.2%) in the TACE + Regorafenib group, 0 (0.0%) in the TACE group (*P* < 0.05); fatigue: 37 (58.7%) in the TACE + Regorafenib group, 10 (14.5%) in the TACE group (*P* < 0.05); hypertension: 19 (30.2%) in the TACE + Regorafenib group, 4 (5.8%) in the TACE group (*P* < 0.05); diarrhea: 15 (23.8%) in the TACE + Regorafenib group, 1 (1.4%) in the TACE group (*P* < 0.05); proteinuria: 6 (9.5%) in the TACE + Regorafenib group, 0 (0.0%) in the TACE group (*P* < 0.05); Overall, TACE + Regorafeni was associated with higher rates of abdominal pain, hand foot reaction, fatigue, hypertension, diarrhea, and proteinuria.

Comparisons between two groups were performed using the chi-square test, and a *P* value < 0.05 was considered to indicate a statistical difference.

### 3.4. Changes in Physical Status and Tumor Marker Levels before and after Treatment in Both Groups

1. Comparison of physical status and tumor marker levels after treatment between the two groups (Table 5);
2. The performance status after treatment in the TACE + Regorafenib group was better than that in the TACE group, with a statistically significant difference (*P* < 0.05). The CEA and CA19-9 negative rates after treatment in the TACE + Regorafenib group were higher than those in the TACE group, with a statistically significant difference (*P* < 0.05). TACE + Regorafenib had higher negative rates of CEA and CA19-9, indicating better efficacy.
3. Compare the changes in physical status and tumor marker levels before and after treatment in each group (Tables 6 and 7).

#### 3.4.1. TACE + Regorafenib group

There was no significant statistical difference in the performance status of patients before and after treatment (*P* > 0.05). The negative rates of CEA and CA19-9 after treatment were higher than those before treatment, with a statistically significant difference (*P* < 0.05).

#### 3.4.2. TACE Group

The performance status of patients after treatment was worse than that before treatment, with a statistical difference (*P* < 0.05). The negative rates of CEA and CA19-9 after treatment were higher than those before treatment, with a statistically significant difference (*P* < 0.05).

The chi-square test was used for comparison between the two groups, and *P* value < 0.05 was statistically different.

### 3.5. Response Evaluation of Tumors after Treatment in the Two Groups

**Figure 2:**
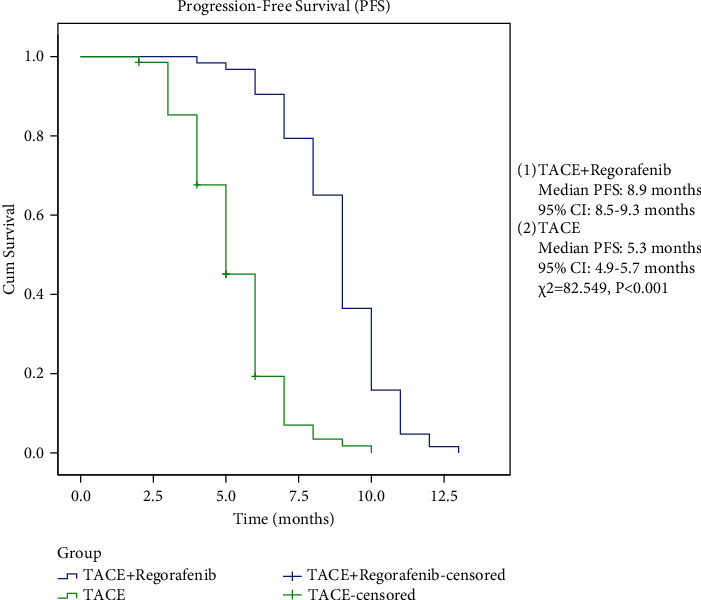
(1) TACE + Regorafenib group, Median OS: 18.2 months (95% CI: 17.1-19.2 months), (2) TACE group Median OS: 11.3 months (95% CI: 10.5-12.1 months), *γ*^2^ = 74.963, *P* < 0.001.

**Figure 3:**
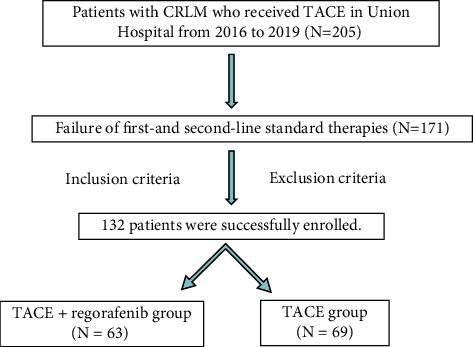
(1) TACE + Regorafenib group. Median PFS: 8.9 months (95% CI: 8.5-9.3 months), (2) TACE group, Median PFS: 5.3 months (95% CI: 4.9-5.7 months), *γ*^2^ = 82.549, *P* < 0.001.

1. Comparison of tumor response between the two groups (Table 8): TACE + Regorafenib group: CR, 8 (12.7%), PR, 28 (44.4%), SD, 16 (25.4%), PD, 11 (17.5%); TACE group: CR, 4 (5.8%), PR, 19 (27.5%), SD, 24 (34.8%), PD, 22 (31.9%); (*P* < 0.05).
2. Comparison of ORR and DCR between the two groups (Table 8): ORR: TACE + Regorafenib group, 36 (57.1%); TACE group, 23 (33.3%); (*P* < 0.05). DCR: TACE + Regorafenib group, 52 (82.5%); TACE group, 47 (68.1%); (*P* < 0.05).
3. Chi-square test was used for comparison between the two groups, and a *P* value < 0.05 was statistically different.
4. Comparison of OS and PFS between the two groups (Table 9, Figures 2 and 3).

OS: mOS 18.2 months (95% CI 17.1–19.2 months) in the TACE + Regorafeni group; mOS 11.3 months (95% CI 10.5–12.1 months) in the TACE group (*P* < 0.05). PFS: mPFS 8.9 months (95% CI 8.5–9.3 months) in the TACE + Regorafenib group; mPFS 5.3 months (95% CI 4.9–5.7 months) in the TACE group (*P* < 0.05).

The log-rank test was used for comparison between the two groups, and a *P* value < 0.05 was statistically significant.

## 4. Discussion

Colorectal cancer is one of the most common malignant tumors, and the incidence is increasing year by year. The liver is the most important target organ of hematogenous metastases of colorectal cancer, and CRLM is one of the main causes of death. Currently, CRLM is commonly classified as resectable, potentially resectable, and unresectable. Although surgical resection is the mainstay of treatment for CRLM, only 15–20% of patients have the opportunity to undergo surgical treatment due to conditions such as lesion size, disease extent, and inadequate liver functional reserve. While for potentially resectable and unresectable patients, the mainstay of treatment remains chemotherapy [28]. Commonly used chemotherapy regimens are FOLFOX, FOLFIRI, FOLFOXIRI, CAPEOX, and so on. The advent of molecularly targeted agents, represented by bevacizumab and cetuximab, has further prolonged the survival of patients with CRLM [29]. Unfortunately, these tumors that initially respond to treatment will eventually progress. Therefore, it is significant to combine effective local therapy in patients with CRLM under the premise of multidisciplinary treatment. Commonly used local treatments for CRLM include ablation therapy [30, 31], I125 seed implantation, transcatheter arterial chemoembolization (TACE) [32], hepatic arteria infusion chemotherapy (HAIC) [33], and TARE [34]. TACE was first reported in the 1970s, and the technique of TACE has been continuously improved with the advent of new devices, drugs, and embolization materials [15]. As one of the commonly used means to treat primary liver cancer, TACE can significantly prolong the OS and PFS of patients [35]. Although the metastatic route of the CRLM is the portal vein, its blood supply comes from the hepatic artery [36]. Therefore, TACE is also a commonly used local treatment for CRLM. C-TACE is performed with lipiodol mixed with chemotherapeutic drugs for embolization, and its efficacy is related to whether the tumor is hypervascular [37]. However, CRLM is hypovascular usually, so the efficacy of C-TACE is sometimes unsatisfactory [37, 38]. In 2006, DEB-TACE was first reported to treat liver metastases [39]. C. Aliberti [39] reported that DEB-TACE (loading irinotecan) was safe and effective in treating patients with CRLMs. Peter Huppert [40] found that TACE (loaded with irinotecan) using SAP microspheres can lead to necrosis of CRLMs, and it is safe and effective. It is reported by An Ngo [41] that fifty-three patients of CRLMs received 125 treatments with DEBIRI (irinotecan-loaded beads), their mOS was 14.5 months, and their mPFS was 5 months. Drug-eluting microspheres used in DEB-TACE are novel embolic agents that slowly and continuously release their preloaded chemotherapeutic agents when injected into the feeding arteries of tumors. Thus, chemotherapeutic drugs can maintain high drug concentrations for a long time in the tumor area. Drug-eluting microspheres can effectively embolize tumor vessels and slowly release chemotherapeutic drugs, so it is very suitable for the chemoembolization of hypovascular tumors. Eichler [42] reported that DEBIRI (irinotecan-loaded DC beads) is a safe treatment for CRLMs with good pharmacokinetics and a high technical success rate. Martin et al. [43] found that, for patients with CRLMs who failed standard treatment, the use of DEBIRI had good efficacy and high safety. Their mPFS was 247 days and their mOS was 343 days from the first treatment. CalliSpheres® drug-loaded microspheres are a new type of embolic agent, which is composed of polyvinyl alcohol, 2-acrylamide, and 2-methylpropanesulfonic acid polymerization and is an ion-exchange embolic microsphere. CalliSpheres® drug-loaded microspheres are negatively charged and can load positively charged chemotherapeutic drugs. Zhao et al. [27] showed that DEBIRI-TACE had a good tumor response and survival benefit in the treatment of unresectable CRLMs, with high tolerance (12-month OS rate: 81.0%; 24-month OS rate: 58.5%; mOS 25.0 months). Irinotecan is positively charged, an analogue of the natural alkaloid camptothecin, and inhibits topoisomerase I. Studies have confirmed that irinotecan loaded DEB-TACE is a safe and effective treatment for CRLM and can be used as a salvage treatment after the failure of first- and second-line therapy [44, 45]. Camillo Aliberti [46] suggested that DEBIRI-TACE could be used as a palliative treatment for unresectable CRLMs who failed chemotherapy. Giammaria Fiorentini [47] reported that DEBIRI-TACE was well tolerated in pretreated patients with CRLMs, and the median length of hospital stay was 3 days. Huang et al. [48] found that for initially unresectable KRAS wild-type CRLMs, chemotherapy combined with TACE improved the negative rate of re-resection in patients and had a better survival benefit compared with chemotherapy alone or chemotherapy plus cetuximab. It is reported by Zacharias et al. [49] that for unresectable CRLMs who failed at least first-line therapy, TACE had a higher OS than others (TACE: 21.3 months vs. HAIC: 13.2 months vs. TARE: 10.7 months). Iezzi et al. [50] suggested that for CRLMs who failed chemotherapy, DEBIRI with capecitabine had a well technical success rate, high safety, and good patient tolerance (mPFS 4 months, mOS 7.3 months). Govindarajan Narayanan [51] found that DEBIRI was well tolerated and safe as a palliative treatment for CRLMs (mOS 13.3, 95% CI: 6.8–19.8 months).

However, the tumor microenvironment will change after TACE. Because hypoxia after embolization induces an increase in VEGF, which can lead to the proliferation of new blood vessels and promote the implantation and growth of circulating tumor cells, resulting in tumor recurrence and metastasis. In order to make up for the lack of TACE, some scholars have tried TACE combined with antiangiogenic drugs in the treatment of CRLMs. Fiorentini et al. [52] found that in the treatment of unresectable CRLMs, the CR rate was 6% and 31%, and the PR rate was 13% and 46% after TACE and TACE-bevacizumab at 3 months; the study showed that TACE-bevacizumab was effective and tolerable. Regorafenib is a drug with indications for the treatment of metastatic colorectal cancer, so TACE combined with regorafenib was chosen in our study. Regorafenib is a multitargeted tyrosine kinase inhibitor [53]. Regorafenib can inhibit a variety of kinases involved in angiogenesis, cell proliferation, and tumor growth as well as the tumor microenvironment, mainly including VEGFR1∼3, KIT, RET, PDGFR-*β*, and FGFR. It can play a role in both the targeted inhibition of tumor cell proliferation and antiangiogenesis [54]. Mechanistically, the combination of TACE and Regorafenib can play a complementary role. TACE combined with targeted drugs has been widely used in the treatment of primary liver cancer, and has achieved very good efficacy. Thomas Walter [11] reported that in the third-line treatment of CRLMs, Regorafenib TAS-102 and SIRT were more effective than the best supportive care. Yamaguchi et al. [55] found that Regorafenib improved the overall survival (mOS was 6.9 months) of patients with CRLM who progress after standard therapies in a real-life setting. Dhillon [56] suggested that Regorafenib was an effective treatment for refractory CRLMs, with controllable adverse events (AEs), which were mostly mild to moderate. Petrioli et al. [57] found that Regorafenib used a modified 2/1 schedule for refractory CRLMs older than 75 years and with poor performance status, which was well tolerated by patients and achieved a good therapeutic effect (mPFS: 4.8 months, mOS: 8.9 months). In a multicenter study (REBECCA) [58], Regorafenib was safe and effective in patients with CRLMs who failed standard treatments. Regorafenib-related AEs occurred in 524 patients (80%), and the most common AEs were poor appetite, asthenia, diarrhea, hand-foot skin reactions, hypertension, and mucositis. The phase-3 trial (CONCUR) [59] showed that for patients with refractory CRLM, the Regorafenib group had a better survival benefit compared with the placebo (mOS, Regorafenib group vs. placebo group: 8.8 months vs. 6.3 months), which is an important option for patients after progression to standard treatment. Tanaka et al. [60] reported that 16 patients with CRLM who failed standard treatment received Regorafenib, with a DCR of 75.0% (95% CI: 50.0–93.8), PFS of 9.0 weeks (8.5–9.5 weeks), and OS of 26.6 weeks (5.0–48.1 weeks).

However, TACE combined with targeted drugs (Regorafenib) is still in the exploratory stage in CRLM. This study found that there was a statistically significant difference in the efficacy evaluation of tumors after TACE + Regorafenib in the TACE group (*P* < 0.05). The ORR and DCR in the TACE + Regorafenib group were significantly better than those in the TACE group. Although the negative rates of serological markers (CEA, CA19-9) after treatment in the TACE + Regorafenib group were increased compared with those before treatment, the CEA negative rate in the TACE + Regorafenib group was significantly higher than that in the TACE group (*P* < 0.05). The OS in the TACE + Regorafenib group (mOS 18.2 months) was higher than that in the TACE group (mOS 11.3 months) (*P* < 0.05). Meanwhile, the PFS in the TACE + Regorafenib group (mPFS 8.9 months) was longer than that in the TACE group (mPFS 5.3 months) (*P* < 0.05). The reason may be that the combination of TACE and Regorafenib plays a synergistic and complementary effect. VEGF levels increased after TACE, and the antiangiogenic effect of Regorafenib compensated for the lack, prolonged the duration of chemoembolization, and inhibited tumor recurrence and metastasis; meanwhile, Regorafenib could inhibit tumor cell proliferation through a variety of pathways. Therefore, the TACE + Regorafenib group was superior to the TACE alone group in both the efficacy evaluation of the tumor and the survival benefit of the patients after treatment. Cao et al. [26] reported that Regorafenib combined with DEB-TACE could achieve better tumor response, PFS, and OS in CRLMs who failed standard treatment compared with Regorafenib monotherapy. Our findings were similar but not identical to them, they compared DEB-TACE + regorafenib with regorafenib alone, and our study compared DEB-TACE + Regorafenib with DEB-TACE alone. The combination of Regorafenib with DEB-TACE was safe and the post-embolization syndrome was tolerable in patients. Kennedy et al. [61] found that the treatment of CRLM using Regorafenib followed by TARE was tolerable (mPFS was 3.7 months and mOS was 12.1 months). Common side effects of TACE include liver function damage and post-embolization syndrome (abdominal pain, fever, nausea, and vomiting). The use of chemotherapeutic agents and arterial embolization leading to liver tissue ischemia can lead to liver function damage. Tissue ischemia after embolization can lead to abdominal pain. Tumor tissue necrosis and inflammatory transmitter release after chemoembolization contribute to patient heating. Chemotherapy agents, tissue ischemia, and TACE procedures can cause nausea and vomiting in patients. Stutz et al. [62] reported that 27 patients with CRLM were treated using DEBIRI, the most common AEs were fatigue (9/27), nausea (8/27), vomiting (6/27), abdominal pain (16/27), and ascites (6/27). He concluded that DEB-TACE is safe in the treatment of CRLMs. Tang et al. [63] found that the common AEs after TACE included hypertension, abdominal pain, fever, nausea, and vomiting. Common adverse reactions of Regorafenib include fatigue, hand-foot reactions, hypertension, proteinuria, diarrhea, and so on. It is reported by Ducreux et al. [64] that the AEs of Regorafenib in the treatment of CRLMs in the real world was the same as those reported in phase III trials, and the most AEs (grade III-IV) included hypertension (6%), fatigue (9%), and hand-foot reactions (7%). Van Cutsem et al. [65] reported that for CRLMs who failed standard therapy (including systemic chemotherapy and monoclonal antibodies targeting VEGF or EGFR), Regorafenib was safe and effective. Kakizawa et al. [66] found that, under the premise of adequate management of patients' AEs, patients with a morphologic response may predict that patients can benefit from Regorafenib treatment. In CONCUR, higher than grade III Regorafenib-related AEs were hand-foot skin reaction (16%), hypertension (11%), hyperbilirubinaemi (7%), hypophosphataemia (7%), ALT increased (7%), AST increased (6%), lipase increased (4%), and maculopapular rash (4%). Whether the combination of TACE and Regorafenib will increase the adverse reactions of patients is a concern for many scholars. This study found that ALT, AST, and total bilirubin in the TACE + Regorafenib group increased after treatment compared with those before treatment, but there was no statistical difference in liver function indicators after treatment between the two groups. However, TACE + Regorafenib had more adverse reactions than the TACE group to fatigue, hand-foot reaction, hypertension, proteinuria, and diarrhea (*P* < 0.05). However, there was a significant difference in ECOG score between the TACE + Regorafenib group and the TACE group. The performance status after treatment was better in the TACE + Regorafenib group than in the TACE group. The reason may be that both groups of patients underwent TACE “as needed” rather than “as timed.” Because the tumor control of the TACE + Regorafenib group is better, the patients in this group receive TACE less frequently and the interval of TACE is longer. While TACE is an invasive procedure, it has an impact on the physical, psychological, and performance status of patients. Compared with the effect of adverse reactions of Regorafenib on the physical status of patients, TACE has a greater impact on patients. Therefore, patients may have better performance status if the times of TACE is reduced. The incidence of abdominal pain after treatment was higher in the TACE + Regorafenib group than in the TACE group (*P* < 0.05). The reason maybe that due to the antiangiogenic effect of Regorafenib, the artery is thinner in the TACE + Regorafenib group, and the ischemic symptoms of patients are more obvious after TACE, so the incidence of abdominal pain is higher.

## 5. Conclusion

For the third-line treatment of CRLM, the combination of TACE + Regorafenib had better tumor response, OS, and PFS than TACE alone. Although the TACE + Regorafenib combination group had slightly more adverse reactions, the adverse reactions were mild and tolerable. Patients in the TACE + Regorafenib group had better performance status and quality of life. Therefore, TACE + Regorafenib combination could be considered as salvage therapy for CRLM who failed the first- and second-line standard therapy.

This is work on a small subset of one study. The shortcomings of this study are that the data are derived from a single center, and it is a retrospective study with limited sample size. Multicenter, large-sample, prospective studies can be carried out at a later stage to provide more help for clinical work.

## Figures and Tables

**Table 1:** Comparison of baseline data before treatment between the two groups.

			Group	
			TACE + Regorafenib (*N* = 63)	TACE (*N* = 69)	Chi-squaretest (*P* value)	*t*-test(*P* value)
Gender	Female	Count (%)	24 (38.1%)	29 (42.0%)	0.723	
	Male	Count (%)	39 (61.9%)	40 (58.0%)		

Child-pugh classification of liver function	A	Count (%)	47 (74.6%)	45 (65.2%)	0.261	
	B	Count (%)	16 (25.4%)	24 (34.8%)		

ECOG	0	Count (%)	14 (22.2%)	16 (23.2%)	0.955	
	1	Count (%)	39 (61.9%)	41 (59.4%)		
	2	Count (%)	10 (15.9%)	12 (17.4%)		

CEA	<5 ug/L	Count (%)	23 (36.5%)	22 (31.9%)	0.587	
	≥5 ug/L	Count (%)	40 (63.5%)	47 (68.1%)		

CA19-9	<37 U/ml	Count (%)	34 (54.0%)	37 (53.6%)	0.854	
	≥37 U/ml	Count (%)	29 (46.0%)	32 (46.4%)		

Primary tumor site	Colon	Count (%)	35 (55.6%)	40 (58.0%)	0.861	
	Rectum	Count (%)	28 (44.4%)	29 (42.0%)		

Surgical excision of primary tumor	No	Count (%)	19 (30.2%)	20 (29.0%)	0.715	
	Yes	Count (%)	44 (69.8%)	49 (71.0%)		

Number of intrahepatic lesions	Single	Count (%)	13 (20.6%)	18 (26.1%)	0.540	
	Multiple	Count (%)	50 (79.4%)	51 (73.9%)		

Maximum diameter of intrahepatic lesions	<3 cm	Count (%)	11 (17.5%)	14 (20.3%)	0.655	
	3–5 cm	Count (%)	20 (31.7%)	17 (24.6%)		
	>5 cm	Count (%)	32 (50.8%)	38 (55.1%)		

Age	Mean ± SD		45.5 ± 10.6	47.6 ± 12.3		0.310

ALT before treatment	Mean ± SD		38.3 ± 8.4	39.2 ± 10.5		0.588

AST before treatment	Mean ± SD		34.2 ± 9.7	32.7 ± 11.6		0.424

TBIL before treatment	Mean ± SD		15.8 ± 5.6	14.1 ± 4.5		0.130

**Table 2:** Comparison of liver function after treatment between the two groups.

	Group	Mean	Std. deviation	*t*-test (*P* value)
ALT after treatment	TACE + Regorafenib	50.4	23.2	0.388
	TACE	47.2	18.7	

AST after treatment	TACE + Regorafenib	47.5	17.9	0.744
	TACE	46.6	11.0	

TBIL after treatment	TACE + Regorafenib	19.1	4.8	0.307
	TACE	19.9	4.1	

**Table 3:** Comparison of liver function before and after treatment between the two groups.

		TACE + Regorafenib			TACE		
		Mean	Std. deviation	Paired samples test (*p*-value)	Mean	Std. deviation	Paired samples test (*P* value)
Pair 1	ALT before treatment	38.3	8.4	0.009	39.2	10.5	0.003
	ALT after treatment	50.4	23.2		47.2	18.7	

Pair 2	AST before treatment	34.2	9.7	0.005	32.7	11.6	0.012
	AST after treatment	47.5	17.9		46.6	11.0	

Pair 3	TBIL before treatment	15.8	5.6	0.021	14.1	4.5	0.028
	TBIL after treatment	19.1	4.8		19.9	4.1	

**Table 4:** Incidence of adverse events after treatment.

			Group		Chi-squaretest (*P* value)
			TACE + Regorafenib (*N* = 63)	TACE (*N* = 69)	Fisher's exact test
Fever	No	Count (%)	22 (34.9%)	25 (36.2%)	0.675
	Yes	Count (%)	41 (65.1%)	44 (63.8%)	

Nausea and vomiting	No	Count (%)	38 (60.3%)	41 (59.4%)	0.816
	Yes	Count (%)	25 (39.7%)	28 (40.6%)	

Abdominal pain	No	Count (%)	20 (31.7%)	47 (68.1%)	0.003
	Yes	Count (%)	43 (68.3%)	22 (31.9%)	

Hand-foot reaction	No	Count (%)	32 (50.8%)	69 (100.0%)	0.001
	Yes	Count (%)	31 (49.2%)	0 (0.0%)	

Fatigue	No	Count (%)	26 (41.3%)	59 (85.5%)	0.012
	Yes	Count (%)	37 (58.7%)	10 (14.5%)	

Hypertension	No	Count (%)	44 (69.8%)	65 (94.2%)	0.008
	Yes	Count (%)	19 (30.2%)	4 (5.8%)	

Diarrhea	No	Count (%)	48 (76.2%)	68 (98.6%)	0.010
	Yes	Count (%)	15 (23.8%)	1 (1.4%)	

Proteinuria	No	Count (%)	57 (90.5%)	69 (100.0%)	0.016
	Yes	Count (%)	6 (9.5%)	0 (0.0%)	

**Table 5:** Comparison of performance status and tumor markers between the two groups after treatment.

			Group		Chi-square test (*P* value)	
			TACE + Regorafenib (*N* = 63)	TACE (*N* = 69)	Pearson chi-square	Fisher's exact test
ECOG score	0	Count (%)	9 (14.3%)	2 (2.9%)	0.037	
	1	Count (%)	40 (63.5%)	44 (63.8%)		
	2	Count (%)	14 (22.2%)	23 (33.3%)		

CEA	<5 *µ*g/L	Count (%)	51 (81.0%)	45 (65.2%)		0.043
	≥5 *µ*g/L	Count (%)	12 (19.0%)	24 (34.8%)		

CA19-9	<37 U/ml	Count (%)	53 (84.1%)	53 (76.8%)		0.382
	≥37 U/ml	Count (%)	10 (15.9%)	16 (23.2%)		

**Table 6:** Comparison of physical score and tumor markers before and after treatment in the TACE + Regorafenib group.

			Group (*N* = 63)		Chi-square test (*P* value)	
			Before treatment	After treatment	Pearson chi-square	Fisher's exact test
ECOG	0	Count (%)	14 (22.2%)	9 (14.3%)	0.413	
	1	Count (%)	39 (61.9%)	40 (63.5%)		
	2	Count (%)	10 (15.9%)	14 (22.2%)		

CEA	<5 *µ*g/L	Count (%)	23 (36.5%)	51 (81.0%)		0.003
	≥5 *µ*g/L	Count (%)	40 (63.5%)	12 (19.0%)		
CA19-9	<37 U/ml	Count (%)	34 (54.0%)	53 (84.1%)		0.010
	≥37 U/ml	Count (%)	29 (46.0%)	10 (15.9%)		

**Table 7:** Comparison of physical score and tumor markers before and after treatment in the TACE group.

			Group (*N* = 69)		Chi-square test (*P* value)	
			Before treatment	After treatment	Pearson chi square	Fisher's exact test
ECOG	0	Count (%)	16 (23.2%)	2 (2.9%)	0.012	
	1	Count (%)	41 (59.4%)	44 (63.8%)		
	2	Count (%)	12 (17.4%)	23 (33.3%)		

CEA	<5 *µ*g/L	Count (%)	22 (31.9%)	45 (65.2%)		0.013
	≥5 *µ*g/L	Count (%)	47 (68.1%)	24 (34.8%)		

CA19-9	<37 U/ml	Count (%)	37 (53.6%)	53 (76.8%)		0.007
	≥37 U/ml	Count (%)	32 (46.4%)	16 (23.2%)		

**Table 8:** Comparison of tumor response evaluation between the two groups after treatment.

			Group			
			TACE + Regorafenib (*N* = 63)	TACE (*N* = 69)	Chi-squaretest (*P* value)	Fisher's exact test (*P* value)
Response evaluation	CR	Count (%)	8 (12.7%)	4 (5.8%)	0.043	
	PR	Count (%)	28 (44.4%)	19 (27.5%)		
	SD	Count (%)	16 (25.4%)	24 (34.8%)		
	PD	Count (%)	11 (17.5%)	22 (31.9%)		

	ORR	Count (%)	36 (57.1%)	23 (33.3%)		0.022
	DCR	Count (%)	52 (82.5%)	47 (68.1%)		0.019

**Table 9:** Comparison of OS and PFS between the two groups.

	Group	Median			Log-rank (mantel-cox)	
		Estimate	95% confidence interval		Chi-square	*P* value
			Lower bound	Upper bound		
OS	TACE + Regorafenib	18.2	17.1	19.2	74.963	0.011
	TACE	11.3	10.5	12.1		

PFS	TACE + Regorafenib	8.9	8.5	9.3	82.549	0.008
	TACE	5.3	4.9	5.7		

## Data Availability

The datasets used and analysed during the current study are available from the corresponding author on reasonable request.

## Abbreviations

TACE: Transcatheter arterial chemoembolization
CRLMs: Colorectal liver metastases
CR: Complete response
PR: Partial response
SD: Stable disease
PD: Progressive disease
ORR: Objective response rate
DCR: Disease control rate
OS: Overall survival
PFS: Progression-free survival
CEA: Carcinoembryonic antigen
CA19-9: Carbohydrate antigen 19-9
ECOG: Eastern cooperative oncology group
VEGFR: Vascular endothelial growth factor receptor
PDGFR: Platelet-derived growth factor receptor
EGFR: Epidermal growth factor receptor
FGFR: Fibroblast growth factor receptor
ALT: Alanine aminotransferase
AST: Aspartate aminotransferase
TBIL: Total bilirubin
HAIC: Hepaticarteria infusion chemotherapy
TARE: Transarterial radioembolization
FOLFOX: Oxaliplatin + fluorouracil + leucovorin
FOLFIRI: Irinotecan + fluorouracil + leucovorin
FOLFOXIRI: Oxaliplatin + irinotecan + fluorouracil + leucovorin
CAPEOX: Capecitabine + oxaliplatin
C-TACE: Conventional TACE
DEB-TACE: TACE with drug-eluting bead
CT: Computed tomography
MRI: Magnetic resonance imaging
AEs: Adverse events.
